# The Anti-Multidrug-Resistant *Acinetobacter baumannii* Study on 1,3-diamino-7H-pyrrolo[3,2-f]quinazoline Compounds

**DOI:** 10.3390/molecules27238609

**Published:** 2022-12-06

**Authors:** Han Wu, Hongtong Chen, Jungan Zhang, Xinxin Hu, Chunyang Xie, Weiting Cao, Ziqi Zhao, Zengshuo Xiao, Yixin Ren, Luyao Dong, Peiyi Sun, Xuefu You, Xinyi Yang, Wei Hong, Hao Wang

**Affiliations:** 1 School of Pharmacy, Minzu University of China, Beijing 100081, China; 2 Key Laboratory of Ethnomedicine, Minzu University of China, Ministry of Education, Beijing 100081, China; 3 Beijing Key Laboratory of Antimicrobial Agents/Laboratory of Pharmacology, Institute of Medicinal Biotechnology, Chinese Academy of Medical Sciences & Peking Union Medical College, Beijing 100050, China; 4 Institute of National Security, Minzu University of China, Beijing 100081, China; 5 Jingjinji National Center of Technology Innovation, Beijing 100094, China

**Keywords:** *Acinetobacter baumannii*, multidrug resistance, dihydrofolate reductase

## Abstract

As a major public health problem, the prevalence of *Acinetobacter baumannii* (*A. baumannii*) infections in hospitals due to the pathogen’s multiple-antibiotic resistance has attracted extensive attention. We previously reported a series of 1,3-diamino-7H-pyrrolo[3,2-f]quinazoline (PQZ) compounds, which were designed by targeting *Escherichia coli* dihydrofolate reductase (*ec*DHFR), and exhibited potent antibacterial activities. In the current study, based on our molecular-modeling study, it was proposed that PQZ compounds may function as potent *A. baumannii* DHFR (*ab*DHFR)-inhibitors as well, which inspired us to consider their anti-*A. baumannii* abilities. We further found that three PQZ compounds, OYYF-171, -172, and -175, showed significant antibacterial activities against *A. baumannii*, including multidrug-resistant (MDR) strains, which are significantly stronger than the typical DHFR-inhibitor, trimethoprim (TMP), and superior to, or comparable to, the other tested antibacterial agents belonging to β-lactam, aminoglycoside, and quinolone. The significant synergistic effect between the representative compound OYYF-171 and the dihydropteroate synthase (DHPS)-inhibitor sulfamethoxazole (SMZ) was observed in both the microdilution-checkerboard assay and time-killing assay, which indicated that using SMZ in combination with PQZ compounds could help to reduce the required dosage and forestall resistance. Our study shows that PQZ is a promising scaffold for the further development of folate-metabolism inhibitors against MDR *A. baumannii*.

## 1. Introduction

The emergence of multidrug-resistant (MDR) pathogenic bacteria, which means the microorganisms are not susceptible to at least one agent in three or more antimicrobial categories, is a global threat to human health [1]. In February 2017, the World Health Organization (WHO) issued its list of pathogens in urgent need of developing new antibacterial agents, to guide the research into and development of the new antibiotics. In this broad list, the pathogens of ESKAPE (*Enterococcus faecium*, *Staphylococcus aureus*, *Klebsiella pneumoniae*, *Acinetobacter baumannii*, *Pseudomonas aeruginosa*, and *Enterobacteriaceae*) were designated as “priority status”. The acquisition of antimicrobial resistance genes by ESKAPE pathogens has caused the reduction of treatment options for serious infections, and increased the burden of disease and the death rates due to treatment failure [2].

*A. baumannii* is a Gram-negative, aerobic coccobacillus that primarily causes nosocomial infections including skin and soft tissue infections, wound infections, bacteremia, endocarditis, urinary tract infections (UTIs), meningitis, and pneumonia [3]. Infections caused by *A. baumannii* have been considered as a major concern in hospital settings, especially intensive care units (ICUs), because of their non-susceptibility or resistance to a wide range of antibiotics, which often leads to no suitable antimicrobial agent for treatments. Several cross-regional or global surveillance programs of antibiotic resistance in *A. baumannii* infections performed recently showed that approximately 45% of all global *A. baumannii* isolates belong to MDR strains, with the MDR rate exceeding 60% in countries (even 90% in some countries) distributed among Latin America, the Middle East, Africa, and Europe [2]. According to a systematic analysis of the global burden of bacterial antimicrobial resistance in 2019, it was estimated that the average number of global deaths attributable to or associated with MDR *A. baumannii* (MDR-AB) exceeded 100,000 and 400,000, respectively [4]. More seriously, in the context of the current COVID-19 pandemic, the burden of morbidity and mortality caused by MDR-AB in nosocomial infections may constantly increase [5,6]. Therefore, there is an urgent need to find new candidate drugs and understand their mechanisms of action, to deal with the severe problem of *A. baumannii*.

**Figure 1:**
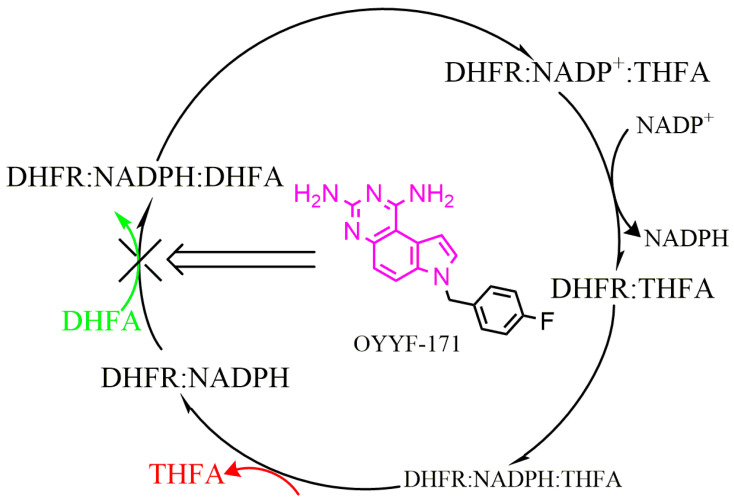
Catalytic cycle of DHFR.

Folates function as crucial cofactors in one-carbon transfers in the biosynthesis of nucleic acids and the metabolism of amino acids, and are necessary for the growth of various cells, including bacteria. Unlike mammalian cells, which can directly obtain folates from dietary sources, bacteria must synthesize folic acid de novo from the inherent substrate, para-amino-benzoic acid (PABA), through the folate pathway catalyzed by multiple enzymes [7]. In the folate pathway, dihydrofolate reductase (DHFR) catalyzes the NADPH-dependent reduction of dihydrofolate (DHFA) to tetrahydrofolate (THFA), and is essential for the subsequent synthesis of thymidylate, purines, and some amino acids (Figure 1) [8]. Inhibition of the enzyme’s activity leads to the arrest of DNA synthesis and to cell death, which has been validated by the utility of classical DHFR inhibitors such as trimethoprim (TMP), and makes it an ideal target for discovering more novel antimicrobials [9].

**Figure 2:**
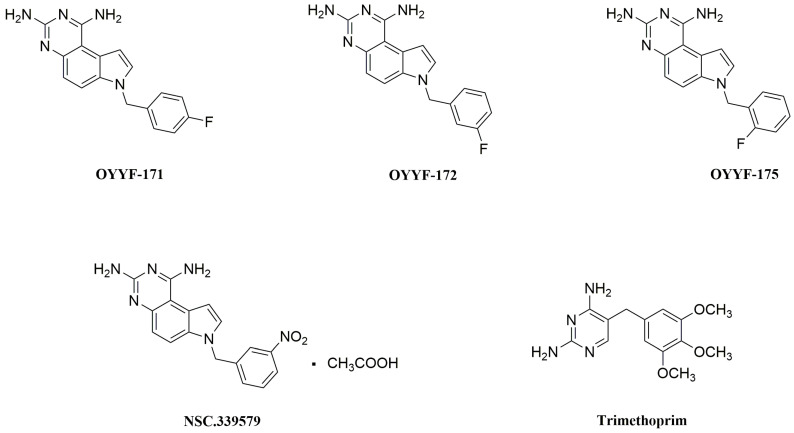
Chemical structures of NSC-339579, OYYF-171, OYYF-172, OYYF-175, and TMP.

We previously reported the discovery of a novel non-classical folate inhibitor, 1,3-diamino-7H-pyrrolo[3,2-f]quinazoline (PQZ) compound (NSC-339579, Figure 2) with strong in vitro inhibitory activity against *Mycobacterium tuberculosis* DHFR (*mt*DHFR, IC_50_ = 6 nM) [10]. Based on the structure of NSC-339579, a series of PQZ compounds were designed and synthesized. Antibacterial studies showed that these compounds exhibited potent broad-spectrum in vitro antibacterial activities [11]. In view of the urgency of developing antimicrobial candidates against *A. baumannii*, especially MDR strains, as described above, we further investigated the in vitro activity of the PQZ compounds OYYF-171, -172, and -175 (Figure 2) against *A. baumannii* including MDR isolates, in this study. Our results indicated that these compounds could be promising leading compounds for further drug design to deal with the serious antibiotic-resistance problem.

## 2. Results

### 2.1. Molecular Modeling

As reported in our previous paper, OYYF-175 has been confirmed as a strong DHFR inhibitor by co-crystallization with *E. coli* DHFR (*ec*DHFR, PDB ID: 7F3B) [7]. With the sequence comparison using BLAST, it was noticed that *ec*DHFR and *A. baumannii* DHFR (*ab*DHFR) share a 54% similar sequence and their DHFA binding sites are highly conserved, which is reasonable because DHFA is the common substrate of both proteins. Since the *ab*DHFR protein structure has not been reported, the homology model was built using Modeller based on *ec*DHFR (PDB ID: 4NX7) [12,13], followed by 50 ns molecular-dynamics simulations to stabilize the conformation.

**Figure 3:**
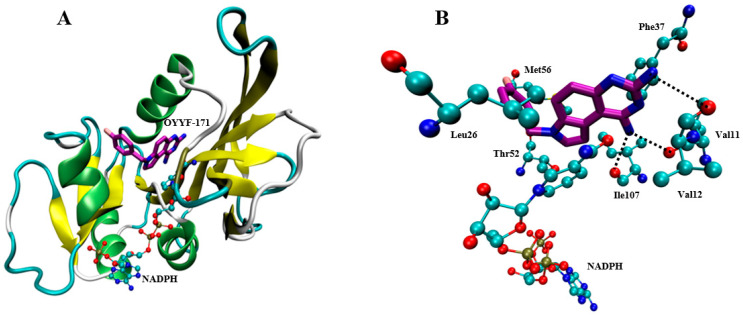
The binding poses of OYYF-171 with *ab*DHFR. In the current figure, the key residues were represented in a ball-and-stick model, in which oxygen atoms were colored red, nitrogen atoms were colored blue, sulfur atoms were colored yellow, and carbon atoms were colored cyan (in protein) or purple (in OYYF-171); the hydrogen bonds were represented by dotted lines. (**A**) The overview of the binding of OYYF-171-NADPH-DHFR. (**B**) The detailed interactions of OYYF-171 with the key residues of homology modeling on *ec*DHFR.

OYYF-171 was docked into *ab*DHFR, and the conformation with the lowest energy was selected as the starting complex for the following 100 ns of molecular-dynamics simulation. During the full simulation, the compound OYYF-171 remained stable in the binding site, and there were no major conformational changes in either protein or ligand. The MM-GBSA and normal mode were performed to calculate the binding free-energy (Table 1) as −15.65 kcal/mol, which indicated a strong binding between OYYF-171 and *ab*DHFR. The 1,3-diamino-7H-pyrrolo[3,2-f]quinazoline part in OYYF-171 forms a stable interaction with the *ab*DHFR, allowing OYYF-171 to be firmly embedded into the protein, and the F-substituted benzyl group points to the solution area and shows a certain degree of flexibility during the simulation. Free-energy decomposition was calculated, and those contributions greater than -1 Kcal/mol were recorded (Figure 3). Among these residues, Leu26, Thr52, and NADPH contributed mainly via van der Waals (vdW) interactions, Phe37 formed π-π interactions with OYYF-171, and Val11, Val12 and Ile107 formed hydrogen bonds with OYYF-171.

Additionally, the molecular-dynamic simulations on the complex of *ab*DHFR with OYYF-172, -175, or TMP were performed, and the binding free-energies were calculated, based on the stable trajectories. In comparison with OYYF-171, although there are some differences on the binding free-energies, the stable and similar global binding-poses of all three compounds were observed. In consideration of the high structural similarities of OYYF-171, -172, and -175, the structure– activity relationships (SAR) of these compounds were carefully analyzed. Based on our simulations, although the global binding-poses of ligands are stable, the F-substituted benzyl groups show certain degrees of freedom, so that the stable interactions between F and any certain amino acids were not observed, which meant, unfortunately, that the SAR cannot be summarized based on the current data.

### 2.2. In Vitro Antibacterial Activity against A. baumannii Isolates

The MIC (Minimum inhibitory concentration) values of OYYF-171, -172, -175, and TMP against eight reference *A. baumannii* strains from ATCC are shown in Table 2. The tested strains include seven MDR-ABs. The PQZ derivatives OYYF-171, -172, and -175 displayed potent antibacterial activities against *A. baumannii*, with MICs of 0.5–16, 1–16, and 1–32 μg/mL, respectively. The antibacterial activities of PQZ compounds were significantly stronger than that of the control drug TMP (MIC = 8–>256 μg/mL).

In order to evaluate and compare the in vitro efficacy of the PQZ compounds and existing antibacterial drugs against *A. baumannii* clinical isolates, a total of 40 strains of *A. baumannii*, including 28 MDR strains from the hospitals in Beijing were further used in MIC determination. The MIC values are shown in Supplementary Table S1, in the material. The MIC_50_s, MIC_90_s, and MIC ranges of three PQZ compounds and ten different agents that belong to four antibiotic classes are shown in Table 3. The MIC ranges of OYYF-171, -172, and -175 were 0.25–32, 0.25–32, and 0.25–>32 µg/mL. The MIC_50_s of three PQZ compounds were lower than that of most of the tested β-lactams, aminoglycoside, quinolones, and folate-metabolism inhibitor, and equivalent to the MIC_50_ of levofloxacin (MIC_50_ = 8 µg/mL). The MIC_50_ values of the three PQZ compounds were only 1/8 of that (MIC_50_ = 128 µg/mL) of the DHFR inhibitor, TMP. The MIC_90_/MIC_50_ of OYYF-171, -172, and -175 were 1, 1, and 2, respectively.

OYYF-171, -172, and -175 showed antibacterial activities against the MDR-AB strains, with MIC ranges of 4–32 μg/mL, 4–32 μg/mL, and 8–>32 μg/mL, respectively. Although these PQZ compounds were generally more active against antibiotic-susceptible *A. baumannii* strains than MDR strains, it was noteworthy that all three compounds were significantly superior to TMP (MIC ≥ 128 μg/mL) in antibacterial activity against the tested MDR-AB strains.

### 2.3. Time-Killing Assay

**Figure 4:**
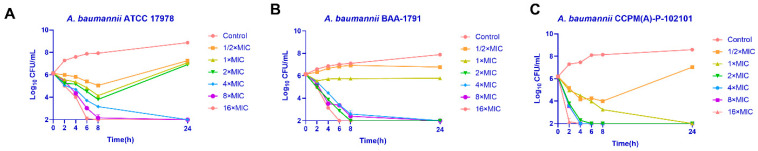
Time-killing curves of OYYF-171 against *A. baumannii* isolates.

A time-killing assay was further conducted to evaluate the dynamic antibacterial characteristics of PQZ OYYF-171 using three *A. baumannii* isolates, including one antibiotic-susceptible strain and two MDR strains (Figure 4). The test concentrations of OYYF-171 were 1/2×, 1×, 2×, 4×, 8× and 16× MIC. The bactericidal effect of the PQZ compound was observed in a time-dependent and widely concentration-dependent manner, which is in consistent with the action modes of most other folate inhibitors. The 3-log_10_ decreases in the number of colony-forming units per mL (CFU/mL) were observed at concentrations of 4–16× MIC, 2–16× MIC and 1–16× MIC within 24 h for *A. baumannii* ATCC 17978, ATCC BAA-1791 and CCPM(A)-P-102101, respectively. The rapid sterilization period can be achieved in 4–8 h.

### 2.4. Synergistic Effect with Sulfamethoxazole (SMZ)

In the folate pathway, DHPS is an essential upstream-enzyme of DHFR, and has been deemed as one of the important antimicrobial targets. Sulfonamides, including SMZ, can specifically inhibit DHPS. To evaluate the synergistic effect that can be achieved by multi-targeting inhibition on the enzymes of the folate pathway, the combination effect of OYYF-171 with SMZ was tested against *A. baumannii* isolates.

In the checkerboard assay, OYYF-171 showed a significant synergistic effect with SMZ against ten tested *A. baumannii* strains. As shown in Table 4, the fractional inhibitory concentration index (FICI) ranges from 0.020 to 0.375 (the FICI median value was 0.185). Most strains we tested showed high-level resistance to SMZ, and the combined SMZ could inhibit the growth of sulfonamide-resistant bacteria at a concentration lower than the susceptibility breakpoint (≤256 μg/mL), indicating that under the action of the PQZ compound, the antibacterial activity of SMZ against the tested *A. baumannii* isolates could be significantly restored at a therapeutically available concentration.

**Figure 5:**
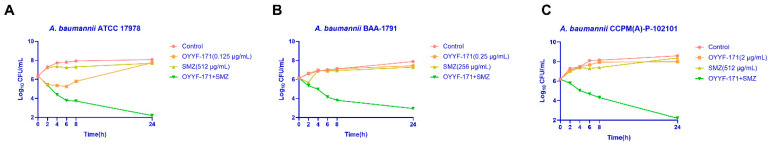
Time-killing curve of SMZ and OYYF-171 used alone and in combination against *A. baumannii* isolates.

The time-killing assay was conducted to further confirm the synergistic antibacterial effect of OYYF-171 and SMZ, using antibiotic-susceptible *A. baumannii* ATCC 17978, MDR-AB ATCC BAA-1791, and CCPM(A)-P-102101. As shown in Figure 5, OYYF-171 and SMZ showed synergetic bactericidal action against three tested *A. baumannii* strains. A ≥2-log_10_ decrease in the number of CFU/mL was observed within 24 h in all three OYYF-171-SMZ combination groups, while no obvious bactericidal effect was observed in SMZ or OYYF-171 monotherapy groups with the same concentrations.

## 3. Discussion

The increasing prevalence of MDR-AB in hospital infections poses a serious threat to the health of patients, which leads to a strong desire for the discovery of new antibacterial agents to defeat the pathogen. In this study, we evaluated the inhibitory/killing activity and characteristics of three PQZ compounds against *A. baumannii*, as well as the synergistic antibacterial effect of one compound, OYYF-171, with SMZ, for the pathogens in vitro.

The PQZ compounds inhibited the growth of *A. baumannii* strains (both antibiotic-susceptible strains and MDR strains). The three PQZs, especially OYYF-171 and -172, were effective in inhibiting the growth of *A. baumannii* strains, and the MIC values against the pathogens were comparable to, or even better than, the reference drugs categorized as β-lactams, aminoglycoside, and quinolones. Our study discovered that although *A. baumannii* is intrinsically resistant to the existing DHFR inhibitor, TMP, it is less resistant to PQZs, which may provide a new idea for developing new DHFR inhibitors.

The use of drug combinations is one of the strategies to prevent the rapid emergence of antibiotic resistance in pathogenic bacteria. The combination of DHFR inhibitors and DHPS inhibitors is one of the most successful examples. Such an advantageous effect is also confirmed in the results of our PQZ-SMZ combination in vitro, and the combination of PQZ with SMZ significantly helps to reduce the MIC of the latter to a susceptible level.

In summary, we identified three PQZ compounds, OYYF-171, -172, and -175 that could serve as the leading compounds for drug development against MDR-AB. Further studies on in vivo efficacy, toxicity, and pharmacodynamics will provide the prospect of improving the leading compounds for more effective antibiotics.

## 4. Materials and Methods

### 4.1. Preparation of Compounds OYYF-171, -172, and -175

The preparation and characterization of OYYF-171, -172, and -175 can be referred to in our previous publication [11].

### 4.2. Molecular Simulation

The homology model *ab*DHFR was built using Modeller based on *ec*DHFR structure (PDB ID: 4NX7) [12]. OYYF-171, -172, -175 and TMP were docked by referring to the binding position of DHFA using GOLD (v5.2; Genetic Optimization for Ligand Docking) [14]. The *ab-initial* quantum mechanics calculations were performed using the B3LYP 6-31G* basis set within Gaussian 16 to optimize the molecular geometries, and the atom-centered point charges were calculated to fit the electrostatic potential, using RESP. The parameterizations of organic molecules were performed with the general AMBER force field (GAFF), using Sobtop [15,16].

Molecular dynamics (MD) simulations were performed with the AMBER99SB-ILDN force field [17] using the GROMACS 2019.6 program [18,19,20]. Each system was first optimized by energy minimizations and equilibrations in line with our standard protocol [10], and followed by a 100 ns free production molecular-dynamic simulation in the NPT ensemble (T = 300 K; P = 1 atm). A total of 100 snapshots were collected from each stable trajectory, and the MM-GBSA and normal mode were performed to calculate the binding free-energy of each molecule. The binding free-energy decomposition was performed, to evaluate the contribution of each residue.

### 4.3. Bacterial Strains and Culture Condition

The reference strains of *A. baumannii* were purchased from the American Type Culture Collection (ATCC) (Manassas, VA, USA). All clinical *A. baumannii* strains were obtained from the Collection Center of Pathogen Microorganisms of the Chinese Academy of Medical Sciences (CAMS-CCPM-A) in China. All isolates were frozen at −80 °C until they were used. Test strains of *A. baumannii* (ATCC and clinical isolates) were picked randomly from fresh cultures on agar plates and resuspended in Mueller Hinton Ⅱ Broth (Cation—Adjusted Mueller Hinton Broth, CAMHB) at 37 °C, with shaking (220 rpm).

### 4.4. Antimicrobial Agents and Susceptibility Test

All antibiotics including ampicillin, ceftazidime, aztreonam, meropenem, ampicillin/sulbactam, gentamicin, ciprofloxacin, levofloxacin, and trimethoprim, were purchased from the National Institutes for Food and Drug Control (Beijing, China). Solvents and diluents for the preparation of antibiotics complied with Clinical and Laboratory Standards Institute (CLSI) guidelines [21].

The MICs of antibiotics and PQZ compounds were determined using the broth-microdilution method, in line with CLSI methodology [21]. All the compounds were serially diluted two-fold across the wells of 96-well standard polystyrene non-treated plates (Corning, Germany), with a final volume of 100 μL per well. Bacteria were cultured in CAMHB at 37 °C, 220 rpm. The bacterial suspension was diluted into CAMHB to get 1 × 10^6^ CFU/mL. A total of 100 μL diluted culture was added to each well of the compound-containing 96-well plates, giving a final concentration of 5 × 10^5^ CFU/mL. All the plates were incubated at 37 °C for 18–20 h. The lowest concentration which showed no visible bacterial growth was defined as the MIC value of the compound. MIC assays were conducted in triplicate for all tested bacterial strains.

### 4.5. Time-Killing Assay

Time-killing assays were performed as describe previously [22]. Bacteria were grown to log phase at 37 °C in CAMHB, and then the bacterial culture was diluted to approximately 2 × 10^6^ CFU/mL, and split into 5 mL volumes. The culture was then mixed with OYYF-171 at concentrations of 1/2× MIC to 16× MIC and returned to incubation at 37 °C, under stationary condition. The starting inoculum was determined from an untreated growth-control at the 0 h time-point, the time at which the culture was split. At 1, 2, 4, 6, 8, and 24 h time-points of incubation, aliquots from each culture were removed. The viability of the cultures over time was monitored by serially diluting samples in sterile saline and spreading 0.1 mL of the diluted samples onto agar plates. The agar plates were incubated overnight at 37 °C in ambient air, and the colony forming units (CFUs) were counted from the plates. Bactericidal activity was defined as a ≥3 log_10_ decrease in the number of viable cells in culture, equivalent to a 99.9% killing of the inoculum. The assay was repeated three times for each of the tested strains.

### 4.6. Synergy Test

The synergy effect of OYYF-171 and SMZ was evaluated through a microdilution-checkerboard assay and time-killing assay [22,23]. The checkerboard assay was assessed on ten *A. baumannii* strains, including drug-sensitive and MDR isolates. In brief, a final inoculum of 5 × 10^5^ CFU/mL was added to wells of 96-well microtiter plates containing two-fold diluted OYYF-171 and SMZ in CAMH broth, and the plates were incubated at 37 °C for 18–20 h. The combined effect of OYYF-171 and SMZ was analyzed by the calculation of the FICI, using the following equation: FICI = (MIC of OYYF-171 in the combination/MIC of OYYF-171 alone) + (MIC of SMZ in the combination/MIC of SMZ alone). The antimicrobial combination was defined as synergistic when the FICI was ≤0.5, additive when 0.5 < FICI ≤ 1, indifferent when 1 < FICI ≤ 2, and antagonistic when FICI > 2. The experiments were performed in duplicate, on different days.

The basic operation of the time-killing assay is the same as above, and 1/4× MIC OYYF-171 and 1/4× MIC SMZ were added alone or in combination, in the test. Synergetic bactericidal action was defined as a ≥2 log_10_ decrease in the number of viable cells in the combination group within 24 h, compared with the initial inoculum concentration and single-drug groups. The assay was repeated three times for each of the tested strain.

## 5. Conclusions

In summary, our study showed that a group of leading compounds (PQZ derivatives) exhibited potential antibacterial activities against both antibiotic-susceptible and MDR isolates of the gram-negative pathogen *A. baumannii*. The antibacterial activities of three PQZ compounds were significantly stronger than that of the reference DHFR inhibitor, TMP. OYYF-171 showed a strong synergistic effect with SMZ for killing the tested *A. baumannii* in vitro, which indicated the potential of PQZ compounds to restore the antibacterial activity of SMZ against *A. baumannii* at a therapeutically available concentration. Further in vivo research and toxicity determination will be carried out.

## Figures and Tables

**Table 1:** Binding free-energies (Kcal/mol) of each compound in complex with *ab*DHFR.

Molecules	ΔG_MM-GBSA_	TΔS	ΔG_binding_
OYYF-171	−30.31 ± 0.34	−11.91 ± 1.66	−18.40
OYYF-172	−35.91 ± 0.85	−13.55 ± 0.55	−22.36
OYYF-175	−34.47 ± 0.74	−17.11 ± 0.04	−17.36
TMP	−35.56 ± 0.35	−10.22 ± 0.77	−25.35

**Table 2:** MICs of OYYF-171, -172, -175, and TMP against ATCC strains of *A. baumannii*.

Strain (Phenotype)		MIC (μg/mL)			
		OYYF-171	OYYF-172	OYYF-175	TMP
*A. baumannii*	ATCC 17978	0.5	1	1	8
	ATCC BAA-1605 (MDR)	2	2	2	64
	ATCC BAA-1789 (MDR)	4	4	8	128
	ATCC BAA-1791 (MDR)	1	1	2	64
	ATCC BAA-1793 (MDR)	8	8	16	128
	ATCC BAA-1794 (MDR)	8	4	8	64
	ATCC BAA-1795 (MDR)	16	16	32	>256
	ATCC BAA-1796 (MDR)	8	8	16	64

MIC, Minimum inhibitory concentration.

**Table 3:** The MIC_50_/MIC_90_ values of PQZ compounds and reference antibiotics against 40 clinical isolates of *A. baumannii*.

Antibiotic		MIC (μg/mL)		
		MIC_50_	MIC_90_	MIC Range
β-lactam	AMP	>32	>32	>32
	CAZ	>32	>32	2–>32
	AZT	64	>64	4–>64
	MEM	>8	>8	0.06–>8
	AMP/SUL	>32	>32	2–>32
Aminoglycoside	GEM	>16	>16	0.5–>16
Quinolone	CIP	>4	>4	0.125–>4
	LEV	8	>8	0.06–>8
Folate metabolism inhibitor	TMP	128	>128	8–>128
PQZ compound	OYYF-171	8	8	0.25–32
	OYYF-172	8	8	0.25–32
	OYYF-175	8	16	0.25–>32

MIC, Minimum inhibitory concentration; AMP, Ampicillin; CAZ, Ceftazidime; AZT, Aztreonam; MEM, Meropenem; AMP/SUL, Ampicillin/Sulbactam; GEM, Gentamicin; CIP, Ciprofloxacin; LEV, Levofloxacin; TMP, Trimethoprim.

**Table 4:** Checkerboard results of the combination of OYYF-171 with SMZ, against *A. baumannii*.

Combination of Drugs	Strain	MIC (µg/mL)				FICI	FICI Median	Checkerboard Effect
		Alone		In Combination				
		OYYF-171	SMZ	OYYF-171	SMZ			
OYYF-171/SMZ	ATCC 17978	0.5	2048	0.125	128	0.313	0.185	Synergism
	ATCC BAA-1791	1	1024	0.25	128	0.375		Synergism
	ATCC BAA-1793	8	1024	0.125	4	0.020		Synergism
	ATCC BAA-1794	8	32	0.125	4	0.141		Synergism
	ATCC BAA-1789	8	2048	1	128	0.188		Synergism
	ATCC BAA-1605	4	2048	0.5	32	0.141		Synergism
	CCPM(A)-P-102101	8	2048	2	256	0.375		Synergism
	CCPM(A)-P-102102	0.25	16	0.03	1	0.183		Synergism
	CCPM(A)-P-102103	8	2048	1	128	0.188		Synergism
	CCPM(A)-P-102105	0.5	1024	0.03	64	0.123		Synergism

MIC, Minimum inhibitory concentration; FICI, Fractional inhibitory concentration index.

## Data Availability

Not applicable.

## Supplementary Materials

The following supporting information can be downloaded at: https://www.mdpi.com/article/10.3390/molecules27238609/s1. Table S1: MICs of OYYF-171, -172, -175 and reference antibiotics against 40 clinical isolates of *A. baumannii*, Tables S2–S4: Colony counts of three *A. baumannii* isolates exposed to OYYF-171 (Log_10_ CFU/mL), Tables S5–S7: Colony counts of three *A. baumannii* isolates exposed to 1/4× MIC OYYF-171/SMZ (Log_10_ CFU/mL).

## Sample Availability

Samples of the compounds are available from the authors.
