# Supplementary material for: The Anti-Multidrug-Resistant Acinetobacter baumannii Study on 1,3-diamino-7H-pyrrolo[3,2-f]quinazoline Compounds
Source: Molecules. 2022 Dec 6;27(23):8609. doi: 10.3390/molecules27238609 (PMC9735644; doi:10.3390/molecules27238609)
Supplement: Supplementary file 1 [file molecules-27-08609-s001.zip › molecules-2064867-supplementary.pdf]

Supplementary material

Evaluation of *in vitro* antibacterial activities of OYYF-171, -172, and -175

Supplementary Table S1. MICs of OYYF-171, -172, -175 and reference antibiotics against 40 clinical isolates of *A. baumannii*

Strain No.	MIC (μg/mL)											
	β-lactam					Aminoglycoside	Quinolone		Folate metabolism inhibitor	PQZ Compound		
	AMP	CAZ	AZT	MEM	AMP /SUL	GEM	CIP	LEV	TMP	OYYF -171	OYYF -172	OYYF -175
CCPM(A)-P-102101	>32	>32	32	>8	>32	>16	>4	8	>128	8	8	8
CCPM(A)-P-102102	>32	4	32	0.5	4	0.5	0.125	0.125	16	0.25	0.25	0.5
CCPM(A)-P-102103	>32	>32	64	>8	>32	>16	>4	>8	>128	8	4	8
CCPM(A)-P-102105	>32	2	8	0.25	2	1	0.125	0.06	16	0.5	0.5	0.5
CCPM(A)-P-102106	>32	>32	64	>8	>32	>16	>4	8	128	8	8	8
CCPM(A)-P-102107	>32	>32	64	>8	>32	>16	>4	8	128	8	8	8
CCPM(A)-P-102108	>32	>32	>64	>8	>32	>16	>4	>8	>128	8	8	16
CCPM(A)-P-102109	>32	>32	64	>8	>32	4	>4	>8	>128	8	8	8
CCPM(A)-P-102110	>32	4	4	0.5	2	>16	2	0.5	32	2	2	2
CCPM(A)-P-102111	>32	>32	64	>8	>32	>16	>4	>8	>128	8	8	8
CCPM(A)-P-102112	>32	>32	64	>8	>32	>16	>4	8	>128	8	8	8
CCPM(A)-P-102113	>32	4	32	0.25	4	1	0.25	0.06	16	0.25	0.25	0.5
CCPM(A)-P-102114	>32	>32	64	>8	>32	>16	>4	8	128	8	8	8
CCPM(A)-P-102115	>32	>32	64	>8	>32	>16	>4	>8	>128	8	8	8
CCPM(A)-P-102116	>32	2	16	0.25	4	0.5	0.125	0.125	8	0.5	0.5	1
CCPM(A)-P-102117	>32	>32	64	>8	>32	>16	>4	>8	>128	8	4	8
CCPM(A)-P-102118	>32	>32	64	>8	>32	>16	>4	>8	>128	8	8	16

CCPM(A)-P-102119	>32	8	16	0.5	4	16	0.5	0.25	16	1	0.5	2
CCPM(A)-P-102120	>32	4	16	0.5	4	8	0.5	0.25	16	2	2	2
CCPM(A)-P-102121	>32	>32	64	>8	>32	>16	>4	8	>128	8	8	8
CCPM(A)-P-102122	>32	4	16	0.25	4	4	0.25	0.125	16	0.5	0.5	2
CCPM(A)-P-102123	>32	2	16	0.5	4	1	0.25	0.125	8	0.25	0.25	0.25
CCPM(A)-P-102124	>32	2	8	0.125	4	0.5	0.125	0.06	8	0.25	0.5	2
CCPM(A)-P-102125	>32	4	32	0.5	4	0.5	0.125	0.125	16	0.25	0.25	0.5
CCPM(A)-P-102126	>32	4	16	1	4	0.5	0.125	0.125	16	0.5	0.25	0.5
CCPM(A)-P-102127	>32	>32	64	>8	>32	>16	>4	>8	128	8	8	8
CCPM(A)-P-102128	>32	>32	64	>8	>32	>16	>4	8	>128	8	8	8
CCPM(A)-P-102129	>32	>32	>64	>8	>32	>16	>4	>8	>128	8	8	16
CCPM(A)-P-102130	>32	>32	64	>8	>32	>16	>4	>8	>128	8	8	16
CCPM(A)-P-102131	>32	>32	>64	>8	>32	>16	>4	>8	>128	8	8	16
CCPM(A)-P-102132	>32	>32	64	>8	>32	>16	>4	>8	128	8	8	16
CCPM(A)-P-102134	>32	>32	>64	>8	>32	>16	>4	>8	>128	32	32	>32
CCPM(A)-P-102136	>32	32	64	0.06	>32	>16	>4	>8	>128	32	32	>32
CCPM(A)-P-102137	>32	>32	64	>8	>32	>16	>4	8	>128	8	8	16
CCPM(A)-P-102166	>32	>32	>64	>8	>32	>16	>4	8	128	8	8	8
CCPM(A)-P-102167	>32	>32	>64	>8	>32	4	>4	>8	>128	16	16	16
CCPM(A)-P-102176	>32	>32	64	>8	>32	>16	>4	8	128	8	8	8
CCPM(A)-P-102180	>32	>32	64	>8	>32	>16	>4	8	128	8	8	8
CCPM(A)-P-102182	>32	16	32	>8	>32	>16	>4	>8	>128	8	8	16

CCPM(A)-P-102183	>32	>32	64	>8	>32	>16	>4	8	128	4	8	8

AMP, Ampicillin; CAZ, Ceftazidime; AZT, Aztreonam; MEM, Meropenem; AMP/SUL, Ampicillin/Sulbactam; GEM, Gentamicin; CIP, Ciprofloxacin; LEV, Levofloxacin; TMP, Trimethoprim

Light gray shading: MDR isolates

Supplementary Table S2. Colony counts of *A.baumannii* ATCC 17978 exposed to OYYF-171 (Log_{10} CFU/mL)

Time（hour）	Concentration						
	0	1/2×MIC	1×MIC	2×MIC	4×MIC	8×MIC	16×MIC
0	6.13	6.13	6.13	6.13	6.13	6.13	6.13
2	7.29	5.99	5.57	5.24	5.06	5.45	5.03
4	7.60	5.83	5.37	5.18	4.69	4.35	4.01
6	7.88	5.41	4.85	4.52	3.72	3.04	2.10
8	7.93	5.05	4.15	3.81	3.17	2.16	2.00
24	8.87	7.26	7.08	6.91	2.00	2.00	2.00

Supplementary Table S3. Colony counts of *A. baumannii* ATCC BAA-1791 exposed to OYYF-171 (Log_{10} CFU/mL)

Time（hour）	Concentration						
	0	1/2×MIC	1×MIC	2×MIC	4×MIC	8×MIC	16×MIC
0	6.15	6.15	6.15	6.15	6.15	6.15	6.15
2	6.60	6.33	5.56	4.97	5.45	5.31	4.98
4	6.87	6.67	5.72	3.89	4.47	3.53	3.14
6	7.03	6.84	5.75	2.89	3.42	3.37	2.00
8	7.11	6.93	5.74	2.00	2.60	2.39	2.00
24	7.89	6.78	5.79	2.00	2.00	2.00	2.00

Supplementary Table S4. Colony counts of *A. baumannii* CCPM(A)-P-102101 exposed to OYYF-171 (Log_{10} CFU/mL)

Time（hour）	Concentration						
	0	1/2×MIC	1×MIC	2×MIC	4×MIC	8×MIC	16×MIC
0	6.19	6.19	6.19	6.19	6.19	6.19	6.19
2	7.29	5.18	4.97	3.79	3.59	3.54	2.16
4	7.46	4.16	4.50	2.30	2.00	2.00	2.00
6	8.11	4.25	3.97	2.00	2.00	2.00	2.00
8	8.14	4.00	3.24	2.00	2.00	2.00	2.00
24	8.59	7.03	2.00	2.00	2.00	2.00	2.00

Supplementary Table S5. Colony counts of *A. baumannii* ATCC 17978 exposed to 1/4×MIC OYYF-171/SMZ (Log_{10} CFU/mL)

Time (hour)	Drug			
	Control	OYYF-171	SMZ	OYYF-171+SMZ
0	6.36	6.36	6.36	6.36
2	7.31	5.45	7.27	5.40

4	7.74	5.36	7.35	4.39
6	7.80	5.26	7.24	3.78
8	7.94	5.80	7.33	3.72
24	8.09	7.74	7.70	2.20

Supplementary Table S6. Colony counts of *A. baumannii* ATCC BAA-1791 exposed to 1/4×MIC OYYF-171/SMZ (Log_{10} CFU/mL)

Time (hour)	Drug			
	Control	OYYF-171	SMZ	OYYF-171+SMZ
0	6.15	6.15	6.15	6.15
2	6.60	6.65	5.70	5.35
4	6.87	6.96	6.96	4.98
6	7.03	6.94	6.89	4.16
8	7.11	7.13	6.94	3.81
24	7.89	7.45	7.28	2.95

Supplementary Table S7. Colony counts of *A. baumannii* CCPM(A)-P-102101 exposed to 1/4×MIC OYYF-171/SMZ (Log_{10} CFU/mL)

Time (hour)	Drug			
	Control	OYYF-171	SMZ	OYYF-171+SMZ
0	6.19	6.19	6.19	6.19
2	7.29	7.08	7.00	5.78
4	7.46	7.47	7.37	5.03
6	8.11	7.67	7.29	4.68
8	8.14	7.94	7.39	4.30
24	8.59	8.00	8.37	2.20
